# 1′-Methyl-4′-(1-naphth­yl)-1′′,2′′,3′′,4′′-tetra­hydro­indane-2-spiro-2′-pyrrolidine-3′-spiro-2′′-naphthalene-1,3,1′′-trione

**DOI:** 10.1107/S1600536811004880

**Published:** 2011-02-12

**Authors:** S. Selvanayagam, B. Sridhar, K. Ravikumar, P. Saravanan, R. Raghunathan

**Affiliations:** a Department of Physics, Kalasalingam University, Krishnankoil 626 190, India; b Laboratory of X-ray Crystallography, Indian Institute of Chemical Technology, Hyderabad 500 007, India; c Department of Organic Chemistry, University of Madras, Guindy Campus, Chennai 600 025, India

## Abstract

In the title compound, C_32_H_25_NO_3_, the pyrrolidine ring adopts an envelope conformation, whereas the cyclo­hexa­none ring in the tetra­hydro­naphthalene fused-ring system adopts a half-chair conformation. The indanedione unit is oriented at an angle of 58.9 (1)° with respect to the naphthyl ring system. Three intra­molecular C—H⋯O close contacts and an intra­molecular C—H⋯π inter­action are observed. In the crystal, mol­ecules associate *via* C—H⋯O hydrogen bonds, forming a helical chain with a *C*(10) motif along the b axis.

## Related literature

For general background to pyrrolidine derivatives, see: Bello *et al.* (2010 ▶); Pettersson *et al.* (2011 ▶). For related structures, see: Abdul Ajees *et al.* (2002 ▶); Selvanayagam *et al.* (2005 ▶). For ring puckering parameters, see: Cremer & Pople (1975 ▶); Nardelli (1983 ▶).
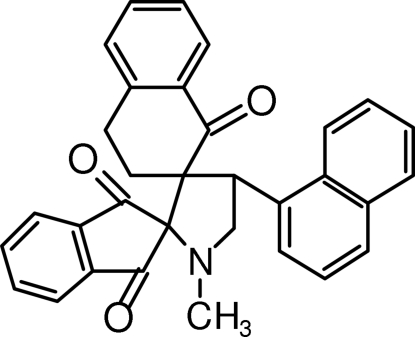


## Experimental

### 

#### Crystal data

C_32_H_25_NO_3_
*M* *_r_* = 471.53
Orthorhombic, 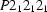

*a* = 10.8442 (9) Å
*b* = 11.431 (1) Å
*c* = 19.2701 (16) Å
*V* = 2388.7 (3) Å^3^
*Z* = 4
Mo *K*α radiation
μ = 0.08 mm^−1^
*T* = 292 K
0.24 × 0.22 × 0.20 mm

#### Data collection

Bruker SMART APEX CCD area-detector diffractometer
28122 measured reflections
3233 independent reflections
2941 reflections with *I* > 2σ(*I*)
*R*_int_ = 0.025

#### Refinement

*R*[*F* ^2^ > 2σ(*F* ^2^)] = 0.039
*wR*(*F* ^2^) = 0.112
*S* = 1.10
3233 reflections
326 parameters
H-atom parameters constrained
Δρ_max_ = 0.23 e Å^−3^
Δρ_min_ = −0.16 e Å^−3^

### 

Data collection: *SMART* (Bruker, 2001 ▶); cell refinement: *SAINT* (Bruker, 2001 ▶); data reduction: *SAINT*; program(s) used to solve structure: *SHELXS97* (Sheldrick, 2008 ▶); program(s) used to refine structure: *SHELXL97* (Sheldrick, 2008 ▶); molecular graphics: *ORTEP-3* (Farrugia, 1997 ▶) and *PLATON* (Spek, 2009 ▶); software used to prepare material for publication: *SHELXL97* and *PLATON*.

## Supplementary Material

Crystal structure: contains datablocks I, global. DOI: 10.1107/S1600536811004880/ng5116sup1.cif

Structure factors: contains datablocks I. DOI: 10.1107/S1600536811004880/ng5116Isup2.hkl

Additional supplementary materials:  crystallographic information; 3D view; checkCIF report

## Figures and Tables

**Table 1:** Hydrogen-bond geometry (Å, °) *Cg*1 is centroid of the C1/C5/C6/C11/C12 ring.

*D*—H⋯*A*	*D*—H	H⋯*A*	*D*⋯*A*	*D*—H⋯*A*
C3—H3⋯O3	0.98	2.23	2.772 (2)	114
C4—H4*A*⋯O1	0.97	2.53	3.072 (3)	115
C13—H13*B*⋯O1	0.97	2.59	3.246 (3)	125
C29—H29⋯O2^i^	0.93	2.40	3.218 (2)	146
C17—H17⋯O1^ii^	0.93	2.55	3.442 (3)	163
C14—H14*B*⋯*Cg*1	0.97	2.51	3.146 (2)	123

Symmetry codes: (i) 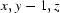; (ii) 
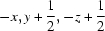
.

**Fig. 1.**
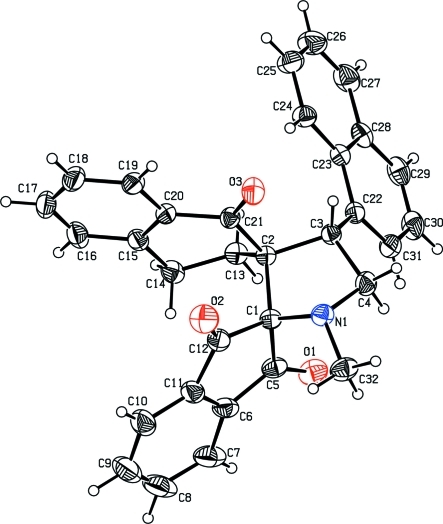
The molecular structure of the title compound, showing the atom-numbering scheme. Displacement ellipsoids are drawn at the 30% probability level

**Fig. 2.**
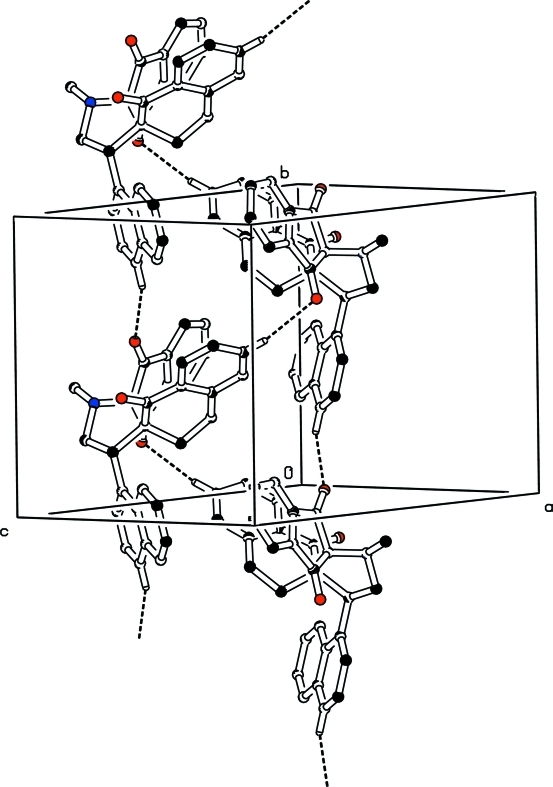
Molecular packing of the title compound, viewed along the a axis; H-bonds are shown as dashed lines. For the sake of clarity, H atoms, not involved in hydrogen bonds, have been omitted

## supplementary crystallographic information

### Comment

Pyrrolidine derivatives are used as norepinephrine reuptake inhibitors and 5-HT(1A) partial agonists for treating neuropsychiatric disorders including depression and anxiety (Pettersson *et al.*, 2011). These derivatives are used as alpha-mannosidase inhibitors and with antitumor activities against hematological and solid malignancies (Bello *et al.*, 2010). In view of these importance, we have undertaken the crystal structure determination of the title compound, a pyrrolidine derivative, and the results are presented here.

The molecular structure of (I) is illustrated in Fig. 1. The C—C bond lengths in the pyrrolidine ring are somewhat longer in particular at two spiro junctions C1 and C2 and the C—N bond lengths are somewhat shorter than normal values. This may be due to steric forces of the bulky substituents at atoms C1 and C2 of the pyrrolidine ring (Abdul Ajees *et al.*, 2002; Selvanayagam *et al.*, 2005).

The sum of the angles at N1 of the pyrrolidine ring [336.3°] is in accordance with sp^3^ hybridization. The short contacts H3···H24 (2.12 Å) and H4A···H31 (1.96 Å) result in substantial widening of the C24—C23—C22 and C31—C22—C3 bond angles [123.8 (2)° and 122.4 (2)°, respectively].

The indanedione moiety is planar with a maximum deviation of 0.051 (3) Å for atom C9. The keto O atoms O1 and O2 deviate from this system by 0.115 (2) and 0.177 (2) Å, respectively. The naphthyl group is also planar with a maximum deviation of 0.013 (2) Å for atom C30. This group is oriented at an angle of 58.9° with respect to the indanedione moiety.

Pyrrolidine ring is in an envelope conformation, with puckering parameters q_2_ = 0.406 (2) Å and φ = 5.3 (2) °, and with atom N1 deviating 0.601 (2) Å from the least-squares plane passing through the remaining four atoms (C1-C4) of that ring (Cremer & Pople, 1975). The cyclohexanone ring in the tetrahydro naphthalin ring system has a half-chair conformation with the lowest asymmetry parameters of ΔC_2_(C2-C13) = 0.070 (1)° (Nardelli, 1983).

The molecular structure is influenced by an intramolecular C—H···O hydrogen bonds and weak C—H···π interactions. In the molecular packing, C—H···O hydrogen bonds involving atoms C17 and O1 link symmetry related molecules to form a helical shape arrangement in the unit cell (Fig. 2 and Table 1). In addition to this another C—H···O hydrogen bonds form a C(10) chain motif in the unit cell.

### Experimental

To a mixture of ninhydrin (1mmol), sarcosine (1mmol) and 2-napthalidene- 1,2,3,4-tetrahydronaphthalene-1-ones (1mmol) was added and heated under reflux in methanol (20ml) until the disappearance of the starting materials as evidenced by TLC. The solvent was removed under vacuo. The crude product was subjected to column chromatography using petroleum ether-ethyl acetate as eluent. Single crystals were grown by slow evaporation from methanol.

### Refinement

H atoms were placed in idealized positions and allowed to ride on their parent atoms, with C—H distances of 0.93-0.97 Å, and Uiso(H) = 1.5U_eq_(C) for methyl H and Uiso(H) = 1.2U_eq_(C,N) for all other H atoms. Due to the lack of anomalous scatterers the absolute configuration was not determined from the X-ray diffraction data and Friedel pairs were merged. The absolute configuration of (I) is unknown.

### Crystal data

**Table:**

C_32_H_25_NO_3_	*F*(000) = 992
*M**_r_* = 471.53	*D*_x_ = 1.311 Mg m^−^^3^
Orthorhombic, *P*2_1_2_1_2_1_	Mo *K*α radiation, λ = 0.71073 Å
Hall symbol: P 2ac 2ab	Cell parameters from 18128 reflections
*a* = 10.8442 (9) Å	θ = 2.2–27.7°
*b* = 11.431 (1) Å	µ = 0.08 mm^−^^1^
*c* = 19.2701 (16) Å	*T* = 292 K
*V* = 2388.7 (3) Å^3^	Block, colourless
*Z* = 4	0.24 × 0.22 × 0.20 mm

### Data collection

**Table:**

Bruker SMART APEX CCD area-detector diffractometer	2941 reflections with *I* > 2σ(*I*)
Radiation source: fine-focus sealed tube	*R*_int_ = 0.025
graphite	θ_max_ = 28.0°, θ_min_ = 2.1°
ω scans	*h* = −14→14
28122 measured reflections	*k* = −15→14
3233 independent reflections	*l* = −24→25

### Refinement

**Table:**

Refinement on *F*^2^	Primary atom site location: structure-invariant direct methods
Least-squares matrix: full	Secondary atom site location: difference Fourier map
*R*[*F*^2^ > 2σ(*F*^2^)] = 0.039	Hydrogen site location: inferred from neighbouring sites
*wR*(*F*^2^) = 0.112	H-atom parameters constrained
*S* = 1.10	*w* = 1/[σ^2^(*F*_o_^2^) + (0.0796*P*)^2^ + 0.0432*P*] where *P* = (*F*_o_^2^ + 2*F*_c_^2^)/3
3233 reflections	(Δ/σ)_max_ < 0.001
326 parameters	Δρ_max_ = 0.23 e Å^−^^3^
0 restraints	Δρ_min_ = −0.16 e Å^−^^3^

### Special details

**Table:**

Geometry. All esds (except the esd in the dihedral angle between two l.s. planes) are estimated using the full covariance matrix. The cell esds are taken into account individually in the estimation of esds in distances, angles and torsion angles; correlations between esds in cell parameters are only used when they are defined by crystal symmetry. An approximate (isotropic) treatment of cell esds is used for estimating esds involving l.s. planes.
Refinement. Refinement of F^2^ against ALL reflections. The weighted R-factor wR and goodness of fit S are based on F^2^, conventional R-factors R are based on F, with F set to zero for negative F^2^. The threshold expression of F^2^ > 2sigma(F^2^) is used only for calculating R-factors(gt) etc. and is not relevant to the choice of reflections for refinement. R-factors based on F^2^ are statistically about twice as large as those based on F, and R- factors based on ALL data will be even larger.

### Fractional atomic coordinates and isotropic or equivalent isotropic displacement parameters (Å^2^)

**Table:**

	*x*	*y*	*z*	*U*_iso_*/*U*_eq_	
O1	0.39079 (16)	0.65842 (13)	0.26515 (8)	0.0626 (4)	
O2	0.27742 (18)	1.00671 (13)	0.15142 (9)	0.0670 (4)	
O3	0.19107 (14)	0.83352 (13)	0.03036 (7)	0.0548 (4)	
N1	0.42985 (14)	0.79880 (14)	0.13310 (8)	0.0437 (3)	
C1	0.32344 (15)	0.80317 (14)	0.17897 (8)	0.0373 (3)	
C2	0.22397 (15)	0.73033 (13)	0.13771 (8)	0.0344 (3)	
C3	0.30573 (15)	0.64431 (14)	0.09301 (8)	0.0379 (3)	
H3	0.2908	0.6644	0.0443	0.045*	
C4	0.43936 (17)	0.67881 (18)	0.10855 (11)	0.0500 (4)	
H4A	0.4747	0.6285	0.1439	0.060*	
H4B	0.4897	0.6743	0.0670	0.060*	
C5	0.34951 (17)	0.75451 (17)	0.25266 (9)	0.0453 (4)	
C6	0.32235 (18)	0.8468 (2)	0.30327 (9)	0.0526 (5)	
C7	0.3266 (2)	0.8410 (3)	0.37595 (11)	0.0731 (7)	
H7	0.3516	0.7735	0.3989	0.088*	
C8	0.2919 (3)	0.9403 (4)	0.41183 (14)	0.0961 (12)	
H8	0.2931	0.9388	0.4601	0.115*	
C9	0.2557 (3)	1.0410 (4)	0.37891 (19)	0.0943 (11)	
H9	0.2323	1.1056	0.4051	0.113*	
C10	0.2536 (2)	1.0476 (2)	0.30753 (15)	0.0730 (7)	
H10	0.2307	1.1161	0.2849	0.088*	
C11	0.28692 (19)	0.94840 (19)	0.27055 (11)	0.0530 (5)	
C12	0.29105 (18)	0.93236 (16)	0.19442 (10)	0.0463 (4)	
C13	0.12866 (17)	0.66910 (15)	0.18410 (9)	0.0412 (4)	
H13A	0.0767	0.6198	0.1554	0.049*	
H13B	0.1716	0.6188	0.2166	0.049*	
C14	0.04745 (18)	0.75315 (19)	0.22442 (9)	0.0474 (4)	
H14A	−0.0166	0.7092	0.2480	0.057*	
H14B	0.0968	0.7921	0.2595	0.057*	
C15	−0.01132 (16)	0.84350 (17)	0.17879 (9)	0.0451 (4)	
C16	−0.1179 (2)	0.90178 (19)	0.19927 (13)	0.0607 (6)	
H16	−0.1524	0.8854	0.2424	0.073*	
C17	−0.17298 (18)	0.9832 (2)	0.15681 (15)	0.0664 (6)	
H17	−0.2446	1.0208	0.1713	0.080*	
C18	−0.1227 (2)	1.0096 (2)	0.09295 (15)	0.0639 (6)	
H18	−0.1602	1.0648	0.0644	0.077*	
C19	−0.01693 (18)	0.95372 (18)	0.07168 (11)	0.0517 (4)	
H19	0.0173	0.9716	0.0287	0.062*	
C20	0.03943 (16)	0.87038 (16)	0.11414 (9)	0.0417 (4)	
C21	0.15470 (15)	0.81437 (15)	0.08854 (8)	0.0379 (3)	
C22	0.27640 (16)	0.51479 (15)	0.10044 (9)	0.0392 (3)	
C23	0.17262 (16)	0.46728 (15)	0.06386 (8)	0.0394 (3)	
C24	0.09433 (18)	0.53423 (18)	0.02056 (10)	0.0481 (4)	
H24	0.1111	0.6134	0.0144	0.058*	
C25	−0.0049 (2)	0.4864 (2)	−0.01248 (12)	0.0605 (5)	
H25	−0.0552	0.5332	−0.0400	0.073*	
C26	−0.0314 (2)	0.3677 (2)	−0.00510 (14)	0.0702 (6)	
H26	−0.0985	0.3352	−0.0281	0.084*	
C27	0.0406 (2)	0.3001 (2)	0.03549 (13)	0.0656 (6)	
H27	0.0218	0.2211	0.0401	0.079*	
C28	0.1443 (2)	0.34598 (15)	0.07131 (10)	0.0480 (4)	
C29	0.2201 (2)	0.27509 (17)	0.11260 (11)	0.0577 (5)	
H29	0.2024	0.1958	0.1169	0.069*	
C30	0.3187 (2)	0.32053 (19)	0.14624 (12)	0.0600 (5)	
H30	0.3686	0.2725	0.1732	0.072*	
C31	0.34582 (19)	0.44138 (18)	0.14031 (10)	0.0509 (4)	
H31	0.4130	0.4716	0.1644	0.061*	
C32	0.54458 (19)	0.8442 (2)	0.16095 (12)	0.0592 (5)	
H32A	0.5318	0.9224	0.1777	0.089*	
H32B	0.6061	0.8450	0.1251	0.089*	
H32C	0.5718	0.7954	0.1985	0.089*	

### Atomic displacement parameters (Å^2^)

**Table:**

	*U*^11^	*U*^22^	*U*^33^	*U*^12^	*U*^13^	*U*^23^
O1	0.0718 (9)	0.0584 (8)	0.0577 (8)	0.0018 (8)	−0.0114 (7)	0.0189 (7)
O2	0.0855 (12)	0.0406 (7)	0.0750 (10)	0.0010 (8)	−0.0074 (9)	0.0104 (7)
O3	0.0616 (8)	0.0629 (8)	0.0400 (6)	0.0169 (7)	0.0096 (6)	0.0150 (6)
N1	0.0374 (7)	0.0486 (8)	0.0451 (7)	−0.0078 (6)	0.0025 (6)	0.0002 (7)
C1	0.0399 (8)	0.0374 (8)	0.0346 (7)	−0.0028 (7)	−0.0015 (6)	0.0035 (6)
C2	0.0358 (7)	0.0346 (7)	0.0327 (7)	−0.0020 (6)	0.0013 (6)	0.0028 (6)
C3	0.0376 (8)	0.0387 (8)	0.0373 (7)	0.0012 (6)	0.0013 (6)	−0.0007 (6)
C4	0.0375 (8)	0.0540 (10)	0.0584 (10)	−0.0019 (8)	0.0070 (8)	−0.0072 (9)
C5	0.0437 (9)	0.0532 (10)	0.0391 (8)	−0.0092 (8)	−0.0046 (7)	0.0081 (7)
C6	0.0436 (9)	0.0732 (13)	0.0409 (8)	−0.0159 (9)	−0.0009 (7)	−0.0064 (9)
C7	0.0628 (13)	0.115 (2)	0.0416 (9)	−0.0265 (14)	−0.0014 (9)	−0.0075 (12)
C8	0.0746 (17)	0.162 (3)	0.0520 (13)	−0.036 (2)	0.0115 (13)	−0.0458 (19)
C9	0.0712 (17)	0.124 (3)	0.0873 (19)	−0.0207 (19)	0.0137 (15)	−0.062 (2)
C10	0.0568 (12)	0.0728 (14)	0.0895 (17)	−0.0096 (12)	0.0046 (12)	−0.0365 (14)
C11	0.0445 (9)	0.0590 (11)	0.0554 (10)	−0.0092 (9)	0.0025 (8)	−0.0154 (9)
C12	0.0448 (9)	0.0405 (8)	0.0535 (9)	−0.0047 (8)	−0.0013 (8)	−0.0034 (8)
C13	0.0425 (8)	0.0435 (8)	0.0377 (7)	−0.0075 (7)	0.0048 (7)	0.0055 (7)
C14	0.0445 (8)	0.0577 (10)	0.0399 (8)	−0.0105 (8)	0.0119 (7)	−0.0032 (8)
C15	0.0366 (8)	0.0483 (9)	0.0505 (9)	−0.0072 (7)	0.0051 (7)	−0.0137 (8)
C16	0.0459 (10)	0.0619 (12)	0.0744 (14)	−0.0072 (9)	0.0156 (10)	−0.0261 (11)
C17	0.0381 (9)	0.0605 (12)	0.1007 (17)	0.0060 (9)	0.0001 (11)	−0.0292 (13)
C18	0.0488 (10)	0.0517 (11)	0.0913 (17)	0.0112 (9)	−0.0177 (11)	−0.0161 (11)
C19	0.0484 (10)	0.0489 (10)	0.0577 (10)	0.0064 (8)	−0.0114 (8)	−0.0061 (9)
C20	0.0362 (8)	0.0430 (8)	0.0459 (8)	0.0014 (7)	−0.0026 (7)	−0.0069 (7)
C21	0.0363 (7)	0.0403 (8)	0.0370 (7)	0.0020 (7)	0.0021 (6)	0.0024 (6)
C22	0.0406 (8)	0.0386 (8)	0.0383 (7)	0.0046 (7)	0.0025 (7)	−0.0006 (6)
C23	0.0432 (8)	0.0371 (8)	0.0379 (7)	0.0018 (7)	0.0034 (7)	−0.0026 (6)
C24	0.0519 (10)	0.0474 (9)	0.0449 (9)	−0.0006 (8)	−0.0057 (8)	0.0011 (7)
C25	0.0532 (11)	0.0731 (14)	0.0551 (11)	−0.0015 (11)	−0.0117 (9)	−0.0020 (11)
C26	0.0621 (13)	0.0746 (15)	0.0740 (15)	−0.0168 (12)	−0.0070 (11)	−0.0181 (13)
C27	0.0706 (14)	0.0475 (10)	0.0789 (14)	−0.0110 (11)	0.0082 (12)	−0.0151 (11)
C28	0.0580 (11)	0.0361 (8)	0.0501 (9)	0.0004 (8)	0.0097 (8)	−0.0059 (7)
C29	0.0759 (14)	0.0347 (8)	0.0625 (11)	0.0090 (9)	0.0135 (11)	0.0012 (8)
C30	0.0762 (14)	0.0471 (10)	0.0566 (11)	0.0250 (10)	0.0023 (11)	0.0087 (9)
C31	0.0531 (10)	0.0507 (10)	0.0488 (10)	0.0124 (9)	−0.0060 (8)	−0.0002 (8)
C32	0.0446 (9)	0.0717 (13)	0.0613 (11)	−0.0167 (10)	−0.0039 (9)	0.0028 (11)

### Geometric parameters (Å, °)

**Table:**

O1—C5	1.210 (3)	C14—H14B	0.9700
O2—C12	1.196 (2)	C15—C16	1.391 (3)
O3—C21	1.208 (2)	C15—C20	1.396 (3)
N1—C32	1.451 (2)	C16—C17	1.376 (4)
N1—C1	1.454 (2)	C16—H16	0.9300
N1—C4	1.455 (3)	C17—C18	1.379 (4)
C1—C12	1.547 (2)	C17—H17	0.9300
C1—C5	1.551 (2)	C18—C19	1.376 (3)
C1—C2	1.578 (2)	C18—H18	0.9300
C2—C13	1.535 (2)	C19—C20	1.397 (3)
C2—C21	1.544 (2)	C19—H19	0.9300
C2—C3	1.580 (2)	C20—C21	1.489 (2)
C3—C22	1.521 (2)	C22—C31	1.364 (3)
C3—C4	1.531 (2)	C22—C23	1.435 (2)
C3—H3	0.9800	C23—C24	1.415 (3)
C4—H4A	0.9700	C23—C28	1.428 (2)
C4—H4B	0.9700	C24—C25	1.365 (3)
C5—C6	1.466 (3)	C24—H24	0.9300
C6—C11	1.376 (3)	C25—C26	1.394 (4)
C6—C7	1.403 (3)	C25—H25	0.9300
C7—C8	1.381 (5)	C26—C27	1.348 (4)
C7—H7	0.9300	C26—H26	0.9300
C8—C9	1.372 (5)	C27—C28	1.419 (3)
C8—H8	0.9300	C27—H27	0.9300
C9—C10	1.378 (5)	C28—C29	1.402 (3)
C9—H9	0.9300	C29—C30	1.354 (3)
C10—C11	1.387 (3)	C29—H29	0.9300
C10—H10	0.9300	C30—C31	1.417 (3)
C11—C12	1.479 (3)	C30—H30	0.9300
C13—C14	1.517 (3)	C31—H31	0.9300
C13—H13A	0.9700	C32—H32A	0.9600
C13—H13B	0.9700	C32—H32B	0.9600
C14—C15	1.499 (3)	C32—H32C	0.9600
C14—H14A	0.9700		
			
C32—N1—C1	116.31 (15)	C15—C14—H14B	109.1
C32—N1—C4	113.40 (16)	C13—C14—H14B	109.1
C1—N1—C4	106.64 (14)	H14A—C14—H14B	107.8
N1—C1—C12	109.27 (14)	C16—C15—C20	118.4 (2)
N1—C1—C5	113.54 (14)	C16—C15—C14	121.12 (18)
C12—C1—C5	101.98 (14)	C20—C15—C14	120.50 (16)
N1—C1—C2	102.60 (12)	C17—C16—C15	121.1 (2)
C12—C1—C2	116.46 (14)	C17—C16—H16	119.5
C5—C1—C2	113.37 (13)	C15—C16—H16	119.5
C13—C2—C21	108.26 (13)	C16—C17—C18	120.4 (2)
C13—C2—C1	114.05 (12)	C16—C17—H17	119.8
C21—C2—C1	108.27 (12)	C18—C17—H17	119.8
C13—C2—C3	114.30 (13)	C19—C18—C17	119.6 (2)
C21—C2—C3	109.00 (12)	C19—C18—H18	120.2
C1—C2—C3	102.69 (12)	C17—C18—H18	120.2
C22—C3—C4	115.47 (15)	C18—C19—C20	120.5 (2)
C22—C3—C2	115.93 (13)	C18—C19—H19	119.8
C4—C3—C2	105.32 (13)	C20—C19—H19	119.8
C22—C3—H3	106.5	C15—C20—C19	120.02 (17)
C4—C3—H3	106.5	C15—C20—C21	122.15 (17)
C2—C3—H3	106.5	C19—C20—C21	117.83 (16)
N1—C4—C3	103.85 (14)	O3—C21—C20	120.24 (16)
N1—C4—H4A	111.0	O3—C21—C2	121.55 (15)
C3—C4—H4A	111.0	C20—C21—C2	118.21 (14)
N1—C4—H4B	111.0	C31—C22—C23	118.48 (17)
C3—C4—H4B	111.0	C31—C22—C3	122.43 (17)
H4A—C4—H4B	109.0	C23—C22—C3	119.09 (15)
O1—C5—C6	126.50 (17)	C24—C23—C28	117.09 (17)
O1—C5—C1	125.10 (17)	C24—C23—C22	123.75 (16)
C6—C5—C1	108.31 (16)	C28—C23—C22	119.15 (16)
C11—C6—C7	120.4 (2)	C25—C24—C23	122.1 (2)
C11—C6—C5	111.01 (16)	C25—C24—H24	118.9
C7—C6—C5	128.6 (2)	C23—C24—H24	118.9
C8—C7—C6	116.9 (3)	C24—C25—C26	120.3 (2)
C8—C7—H7	121.5	C24—C25—H25	119.9
C6—C7—H7	121.5	C26—C25—H25	119.9
C9—C8—C7	122.4 (3)	C27—C26—C25	119.9 (2)
C9—C8—H8	118.8	C27—C26—H26	120.1
C7—C8—H8	118.8	C25—C26—H26	120.1
C8—C9—C10	120.8 (3)	C26—C27—C28	121.9 (2)
C8—C9—H9	119.6	C26—C27—H27	119.0
C10—C9—H9	119.6	C28—C27—H27	119.0
C9—C10—C11	117.7 (3)	C29—C28—C27	121.79 (19)
C9—C10—H10	121.2	C29—C28—C23	119.48 (19)
C11—C10—H10	121.2	C27—C28—C23	118.72 (19)
C6—C11—C10	121.8 (2)	C30—C29—C28	120.87 (18)
C6—C11—C12	109.95 (17)	C30—C29—H29	119.6
C10—C11—C12	128.2 (2)	C28—C29—H29	119.6
O2—C12—C11	126.53 (19)	C29—C30—C31	119.94 (19)
O2—C12—C1	124.96 (17)	C29—C30—H30	120.0
C11—C12—C1	108.43 (16)	C31—C30—H30	120.0
C14—C13—C2	113.59 (14)	C22—C31—C30	122.0 (2)
C14—C13—H13A	108.8	C22—C31—H31	119.0
C2—C13—H13A	108.8	C30—C31—H31	119.0
C14—C13—H13B	108.8	N1—C32—H32A	109.5
C2—C13—H13B	108.8	N1—C32—H32B	109.5
H13A—C13—H13B	107.7	H32A—C32—H32B	109.5
C15—C14—C13	112.50 (14)	N1—C32—H32C	109.5
C15—C14—H14A	109.1	H32A—C32—H32C	109.5
C13—C14—H14A	109.1	H32B—C32—H32C	109.5
			
C32—N1—C1—C12	64.5 (2)	C5—C1—C12—C11	−5.58 (18)
C4—N1—C1—C12	−167.92 (15)	C2—C1—C12—C11	118.38 (16)
C32—N1—C1—C5	−48.6 (2)	C21—C2—C13—C14	−55.95 (19)
C4—N1—C1—C5	78.99 (18)	C1—C2—C13—C14	64.64 (19)
C32—N1—C1—C2	−171.34 (16)	C3—C2—C13—C14	−177.63 (14)
C4—N1—C1—C2	−43.74 (17)	C2—C13—C14—C15	51.9 (2)
N1—C1—C2—C13	151.52 (14)	C13—C14—C15—C16	157.93 (17)
C12—C1—C2—C13	−89.21 (17)	C13—C14—C15—C20	−21.5 (2)
C5—C1—C2—C13	28.67 (19)	C20—C15—C16—C17	0.6 (3)
N1—C1—C2—C21	−87.90 (15)	C14—C15—C16—C17	−178.84 (18)
C12—C1—C2—C21	31.37 (18)	C15—C16—C17—C18	−0.6 (3)
C5—C1—C2—C21	149.25 (14)	C16—C17—C18—C19	0.1 (3)
N1—C1—C2—C3	27.30 (15)	C17—C18—C19—C20	0.3 (3)
C12—C1—C2—C3	146.57 (14)	C16—C15—C20—C19	−0.2 (3)
C5—C1—C2—C3	−95.55 (15)	C14—C15—C20—C19	179.24 (17)
C13—C2—C3—C22	1.7 (2)	C16—C15—C20—C21	178.65 (16)
C21—C2—C3—C22	−119.53 (15)	C14—C15—C20—C21	−1.9 (3)
C1—C2—C3—C22	125.80 (14)	C18—C19—C20—C15	−0.2 (3)
C13—C2—C3—C4	−127.23 (16)	C18—C19—C20—C21	−179.14 (17)
C21—C2—C3—C4	111.49 (16)	C15—C20—C21—O3	174.69 (18)
C1—C2—C3—C4	−3.18 (16)	C19—C20—C21—O3	−6.4 (3)
C32—N1—C4—C3	171.27 (16)	C15—C20—C21—C2	−4.2 (2)
C1—N1—C4—C3	41.97 (18)	C19—C20—C21—C2	174.68 (16)
C22—C3—C4—N1	−151.38 (14)	C13—C2—C21—O3	−146.97 (17)
C2—C3—C4—N1	−22.13 (18)	C1—C2—C21—O3	88.92 (19)
N1—C1—C5—O1	−54.7 (2)	C3—C2—C21—O3	−22.1 (2)
C12—C1—C5—O1	−172.11 (18)	C13—C2—C21—C20	31.93 (19)
C2—C1—C5—O1	61.9 (2)	C1—C2—C21—C20	−92.19 (16)
N1—C1—C5—C6	122.12 (16)	C3—C2—C21—C20	156.82 (14)
C12—C1—C5—C6	4.71 (18)	C4—C3—C22—C31	22.4 (2)
C2—C1—C5—C6	−121.30 (15)	C2—C3—C22—C31	−101.47 (19)
O1—C5—C6—C11	174.55 (19)	C4—C3—C22—C23	−157.10 (15)
C1—C5—C6—C11	−2.2 (2)	C2—C3—C22—C23	79.05 (19)
O1—C5—C6—C7	−6.6 (3)	C31—C22—C23—C24	−179.52 (17)
C1—C5—C6—C7	176.6 (2)	C3—C22—C23—C24	0.0 (3)
C11—C6—C7—C8	1.1 (3)	C31—C22—C23—C28	1.1 (2)
C5—C6—C7—C8	−177.6 (2)	C3—C22—C23—C28	−179.40 (15)
C6—C7—C8—C9	−0.6 (4)	C28—C23—C24—C25	0.6 (3)
C7—C8—C9—C10	−0.6 (5)	C22—C23—C24—C25	−178.80 (18)
C8—C9—C10—C11	1.3 (4)	C23—C24—C25—C26	−0.9 (3)
C7—C6—C11—C10	−0.5 (3)	C24—C25—C26—C27	0.8 (4)
C5—C6—C11—C10	178.48 (18)	C25—C26—C27—C28	−0.3 (4)
C7—C6—C11—C12	179.53 (19)	C26—C27—C28—C29	−179.0 (2)
C5—C6—C11—C12	−1.5 (2)	C26—C27—C28—C23	−0.1 (3)
C9—C10—C11—C6	−0.7 (4)	C24—C23—C28—C29	178.86 (18)
C9—C10—C11—C12	179.3 (2)	C22—C23—C28—C29	−1.7 (3)
C6—C11—C12—O2	−172.2 (2)	C24—C23—C28—C27	−0.1 (3)
C10—C11—C12—O2	7.8 (4)	C22—C23—C28—C27	179.34 (17)
C6—C11—C12—C1	4.7 (2)	C27—C28—C29—C30	179.9 (2)
C10—C11—C12—C1	−175.3 (2)	C23—C28—C29—C30	0.9 (3)
N1—C1—C12—O2	50.9 (2)	C28—C29—C30—C31	0.4 (3)
C5—C1—C12—O2	171.4 (2)	C23—C22—C31—C30	0.3 (3)
C2—C1—C12—O2	−64.7 (2)	C3—C22—C31—C30	−179.19 (18)
N1—C1—C12—C11	−126.03 (16)	C29—C30—C31—C22	−1.1 (3)

### Hydrogen-bond geometry (Å, °)

**Table:**

Cg1 is centroid of the C1/C5/C6/C11/C12 ring.

**Table:**

*D*—H···*A*	*D*—H	H···*A*	*D*···*A*	*D*—H···*A*
C3—H3···O3	0.98	2.23	2.772 (2)	114
C4—H4A···O1	0.97	2.53	3.072 (3)	115
C13—H13B···O1	0.97	2.59	3.246 (3)	125
C29—H29···O2^i^	0.93	2.40	3.218 (2)	146
C17—H17···O1^ii^	0.93	2.55	3.442 (3)	163
C14—H14B···Cg1	0.97	2.51	3.146 (2)	123

Symmetry codes: (i) *x*, *y*−1, *z*; (ii) −*x*, *y*+1/2, −*z*+1/2.
